# Long-chain fatty acid activates hepatocytes through CD36 mediated oxidative stress

**DOI:** 10.1186/s12944-018-0790-9

**Published:** 2018-07-17

**Authors:** Jun Liu, Ping Yang, Guoqing Zuo, Song He, Wei Tan, Xiaoyu Zhang, Chunxiao Su, Lei Zhao, Li Wei, Yao Chen, Xiongzhong Ruan, Yaxi Chen

**Affiliations:** 1 Centre for Lipid Research & Key Laboratory of Molecular Biology for Infectious Diseases (Ministry of Education), Institute for Viral Hepatitis, Department of Infectious Diseases, The Second Affiliated Hospital, Chongqing Medical University, Chongqing, 400016 China; 2 Department of Gastroenterology, Chongqing Hospital of Traditional Chinese Medicine, Chongqing, 400021 China; 3 Department of Gastroenterology, The Second Affiliated Hospital, Chongqing Medical University, Chongqing, 400010 China; 4 John Moorhead Research Laboratory, Centre for Nephrology, University College London Medical School, Royal Free Campus, University College London, NW3 2PF, London, UK; 5 The Collaborative Innovation Center for Diagnosis and Treatment of Infectious Diseases (CCID), Zhejiang University, Hangzhou, 310058 China

**Keywords:** CD36, LCFA, Hepatocyte activation, Oxidative stress

## Abstract

**Background:**

Accumulating evidence suggests that activated hepatocytes are involved in the deposition of the excess extracellular matrix during liver fibrosis via the epithelial to mesenchymal transition. Lipid accumulation in hepatocytes are implicated in the pathogenesis of chronic liver injury. CD36 is known to mediate long-chain fatty acid (LCFA) uptake and lipid metabolism. However, it is unclear whether LCFA directly promotes hepatocyte activation and the involved mechanisms have not been fully clarified.

**Methods:**

Mice were fed with a high fat diet (HFD) and normal hepatocyte cells (Chang liver cells) were treated with palmitic acid (PA) in vivo and in vitro. Real-time polymerase chain reaction (RT-PCR) and western blotting were used to examine the gene and protein expression of molecules involved in hepatic fibrogenesis and hepatocyte activation. CD36 was knocked down by transfecting CD36 siRNA into hepatocyte cells. Hydrogen peroxide (H_2_O_2_) and reactive oxygen species (ROS) levels were detected using commercial kits.

**Results:**

HFD induced a profibrogenic response and up-regulated CD36 expression in vivo. Analogously, PA increased lipid accumulation and induced human hepatocyte activation in vitro, which was also accompanied by increased CD36 expression. Interestingly, knockdown of CD36 resulted in a reduction of hepatocyte lipid deposition and decreased expression of *Acta2* (34% decrease)*, Vimentin* (29% decrease)*, Desmin* (60% decrease), and TGF-β signaling pathway related genes. In addition, HFD and PA increased the production of H_2_O_2_ in vivo (48% increase) and in vitro (385% increase), and the antioxidant, NAC, ameliorated PA-induced hepatocyte activation. Furthermore, silencing of CD36 in vitro markedly attenuated PA-induced oxidative stress (H_2_O_2_: 41% decrease; ROS: 39% decrease), and the anti-activation effects of CD36 knockdown could be abolished by pretreatment with H_2_O_2_.

**Conclusions:**

Our study demonstrated that LCFA facilitates hepatocyte activation by up-regulating oxidative stress through CD36, which could be an important mechanism in the development of hepatic fibrosis.

## Background

Liver cirrhosis is the end-stage condition of various chronic liver diseases, and fibrosis is the precursor of cirrhosis, which is also considered a severe disease [1, 2]. Liver fibrosis is a reversible wound healing response to liver injury and is considered to be a pathological process characterized by the production and excessive deposition of extracellular matrix (ECM) [3, 4]. Liver fibrogenic cells participate in the process via different mechanisms of the epithelial-mesenchymal transition (EMT) when epithelial cells lose their original characteristics and gradually obtain a mesenchymal phenotype, and this transition can be called activation [5, 6]. Even though activated hepatic stellate cells (HSCs) are the most important source of ECM proteins during the fibrogenesis [7], a wealth of evidence has confirmed that hepatocytes are involved in EMT during hepatic fibrosis, and they have been considered to be another type of myofibroblast-like cells [8]. It is known that activated hepatocytes up-regulate the expression of typical mesenchymal cell markers, such as ɑ-SMA, type 1 collagen, vimentin, desmin, and fibronectin, and down-regulate E-cadherin and cytokeratin [9, 10]. TGF-β is recognized as a potent inducer of EMT and a major cytokine in liver fibrosis that induces the profibrogenic pathway and fibrosis in the liver [11].

Excess lipid accumulation in the liver may be an important cause of pathogenesis in chronic liver injury [12]. Studies have found a high correlation between steatosis and the severity of hepatic fibrosis. The accumulation of abnormally high amounts of lipids in hepatocytes may induce increased susceptibility to secondary injury, leading to an acceleration in the process of chronic liver injury [13]. Increased free cholesterol accumulation in HSCs plays a crucial role in the progression of liver fibrosis by promoting HSC activation, which results in further accumulation of free cholesterol and exaggerates liver fibrosis in a vicious cycle [14]. Furthermore, owing to the emerging role of long-chain fatty acids (LCFA) in fibrosis, Lars et al. proved that LCFA (oleate:palmitic acid (PA) 2:1) had profibrogenic effects on LX-2 via upregulation of a-SMA and TGF-β mRNA expression [15]. However, the role of LCFA in the regulation of hepatocyte activation is unknown.

CD36, which belongs to the class B scavenger receptors, is a transmembrane glycoprotein that serves as a facilitator of lipid transport and binds various lipids, e.g., LCFA and oxidized low-density lipoprotein (ox-LDL) [16]. Previous studies demonstrated that CD36 is involved in various diseases, such as insulin resistance, atherosclerosis, and non-alcoholic fatty liver disease (NAFLD) [17]. Additionally, our recent studies found that CD36 plays an important role in balancing the hepatitis B virus life cycle and hepatic inflammation, which could lead to a new potential therapeutic strategy for the prevention of chronic liver injury [18]. More importantly, Wilhelm et al. demonstrated that ox-LDL stimulated ECM synthesis in cultured HSCs through CD36 [19], suggesting that CD36 plays an important role in the pathogenesis of hepatic fibrogenesis.

The purpose of this study was to characterize and examine the effects of PA, a LCFA, on hepatocyte activation and explore whether CD36 was involved in the potential mechanism. Firstly, we found that PA treatment significantly increased CD36 expression and induced hepatocyte activation. Secondly, PA induced hepatocyte activation depended on the oxidative stress pathway. Thirdly, knockdown of CD36 reduced oxidative stress as well as hepatocyte activation. Based on these data, we suggested that CD36 activation by PA induces hepatocyte activation through a oxidative stress dependent pathway.

## Methods

### Cell culture

The Chang liver cell line was obtained from BeNa Culture Collection and cultured with RPMI-1640 medium containing 10% fetal bovine serum, 100 units/ml penicillin, and 100 μg/ml streptomycin. All experiments were carried out in serum-free RPMI-1640 medium containing 0.2% bovine serum albumin (BSA), 100 units/ml penicillin, and 100 μg/ml streptomycin. The cells were pre-incubated in serum-free medium for 12 h and then subjected to PA for another 48 h. Antioxidants N-acetylcysteine (NAC) and hydrogen peroxide (H_2_O_2_) were obtained from Beyotine Biotechnology (Beijing, China), and PA was obtained from Sigma (Poole, Dorset, UK).

### Animal model

Animal care and experimental procedures were approved by the Animal Care Committees at Chongqing Medical University (Number: 2014056). Six- to eight-week-old C57BL/6 J mice were randomly assigned to receive a normal chow diet (NCD, Research Diets, D12450B, 10 kcal% from Fat, *n* = 5) or a high-fat diet (HFD, Research Diets, D12492, 60 kcal% from fat, n = 5). Finally, the mice were killed after 14 weeks. Blood samples were taken for lipid profiles, and liver samples were collected for further assessments.

### Serum analysis

Serum triglyceride (TG) concentrations were determined enzymatically with commercial kits (Jiancheng, Nanjing, China). Free fatty acid (FFA) concentrations were determined calorimetrically using commercial kits (Applygen Technologies, Beijing, China).

### Cell proliferation assay

Cellular proliferation was assayed using a Cell Counting Kit-8 (CCK-8) purchased from Beyotime Biotechnology (Beijing, China). Briefly, hepatocytes were seeded at a density of 2 × 10^4^ in a 96-well culture plate and treated with or without PA for 48 h. Then, absorbance was measured at 450 nm via a Microplate Reader (Bio-Tek, Vermont, USA).

### Western blotting

The total protein content from hepatocytes or liver was lysed using RIPA containing protease inhibitor, and the protein content was measured and normalized using a BCA Protein Assay Kit. Total proteins (20–30 μg) were separated by SDS-PAGE and transferred onto PVDF membranes. After blocking with 3% BSA, the membranes were incubated with primary antibodies anti-CD36, 1:2000 (Novus, Colorado, USA); anti-a-SMA 1:500 (Sigma, Poole, Dorset, UK); anti-vimentin 1:1000 (CST, Danvers, USA); and anti-β-actin, 1:3000 (ProteinTech, Wuhan, China) at 4 °C overnight and subsequently incubated with their corresponding horseradish peroxidase-labeled secondary antibodies. Finally, the blot was detected using an ECL advance Western Blotting Detection Kit (Millipore, Temecula, CA, USA). The protein relative intensity was analyzed using ImageJ software (National Institutes of Health, USA).

### Real-time reverse transcription polymerase chain reaction

Total RNA was homogenized from cultured cells and livers of C57BL/6 J mice using RNAiso Plus reagent (Takara, Dalian, China). cDNA synthesis and quantitative real-time PCR were performed with commercial kits (Takara, Dalian, China) using the Bio-Rad CFX Connect TM Real-Time System (Bio-Rad, Hercules, CA, USA) according to the manufacturer’s instructions. β-actin served as the reference housekeeping gene in vivo and in vitro. All the primer pairs used in this study are listed in Table 1.

**Table 1:** Primers for quantitative real-time PCR

Gene	Primer sequences
Human CD36	Forward: 5′-CTTTGGCTTAATGAGACTGGGAC-3′ Reverse: 5′- GCAACAAACATCACCACACCA-3′
Human β-actin	Forward: 5′-GTTGTCGACGACGAGCG-3′ Reverse : 5′-GCACAGAGCCTCGCCTT-3′
Human Acta2	Forward: 5′-CATCATGCGTCTGGATCTGG-3′ Reverse : 5′-GGACAATCTCACGCTCAGCA-3′
Human Vimentin	Forward: 5′-ACCCGCACCAACGAGAAGGT-3′ Reverse : 5′-ATTCTGCTGCTCCAGGAAGCG-3′
Human TGF-β	Forward: 5′-AAGTTGGCATGGTAGCCCTT-3′ Reverse: 5′-CCCTGGACACCAACTATTGC-3′
Human Snail2	Forward: 5′-GCAGTGAGGGCAAGAAAAAG-3′ Reverse : 5′-TCGGACCCACACATTACCTT-3′
Human Twist1	Forward: 5′-TCCATTTTCTCCTTCTCTGGAA-3′ Reverse: 5′-CCTTCTCGGTCTGGAGGAT-3′
Human Zeb1	Forward: 5′-CAGTCAGCTGCATCTGTAACAC-3′ Reverse: 5′-CCAGGTGTAAGCGCAGAAAG-3′
Mouse CD36	Forward: 5′-GAGCCATCTTTGAGCCTTCA-3′ Reverse: 5′-TCAGATCCGAACACAGCGTA-3′
Mouse β-actin	Forward: 5′-CGATGCCCTGAGGCTCTTT-3′ Reverse: 5′-TGGATGCCACAGGATTCCAT-3′
Mouse Acta2	Forward:5′-CCAGAGCAAGAGAGGGATCCT-3′ Reverse: 5′-TGTCGTCCCAGTTGGTGA-3′
Mouse Col1	Forward: 5′-CAACCTGGACGCCATCAAG-3′ Reverse: 5′-CAGACGGCTGAGTAGGGAACA-3′
Mouse Col4	Forward:5′-CCGAGCCAGTCCATTTATAGAATG-3′ Reverse: 5′-CAGCGAAGCCAGCCAGAA-3′

### ROS, H_2_O_2,_ and malondialdehyde (MDA) assays

Hepatocyte intracellular ROS and H_2_O_2_ content was measured using the ROS and H_2_O_2_ Assay Kit (Beyotime, Beijing, China) according to the manufacturer’s instructions and was normalized by protein concentration. Hepatic MDA content was evaluated using a commercial kit (Jiancheng, Nanjing, China) and was normalized by total liver protein.

### BODIPY 493/503 staining

BODIPY 493/503 (4, 4- difluoro-1, 3, 5, 7- tetramethyl- 4- bora- 3a, 4a- diaza- s- indacene) is a fluorescent lipophilic stain widely used to label lipid droplets in plants. Briefly, solubilized BODIPY 493/503 in DMSO at 2.5 mg/ml was stored at − 20 °C in the dark. Fixed cells were incubated with a working concentration of 0.2 μg/ml BODIPY staining solution in the dark for 30 min at 37 °C. The cells were washed twice with PBS, and the stained cells were visualized using a fluorescence microscope (Zeiss, Jena, Germany).

### Statistical analysis

The data are expressed as the mean ± SEM. Statistical analysis was performed using Student’s t-test when only two value sets were compared and one-way analysis of variance followed by Turkey’s multiple comparison test when the data involved three or more groups. A difference was considered significant if the *P* was less than 0.05.

## Results

### HFD enhances profibrogenic gene expression, along with increased CD36 expression

**Fig. 1:**
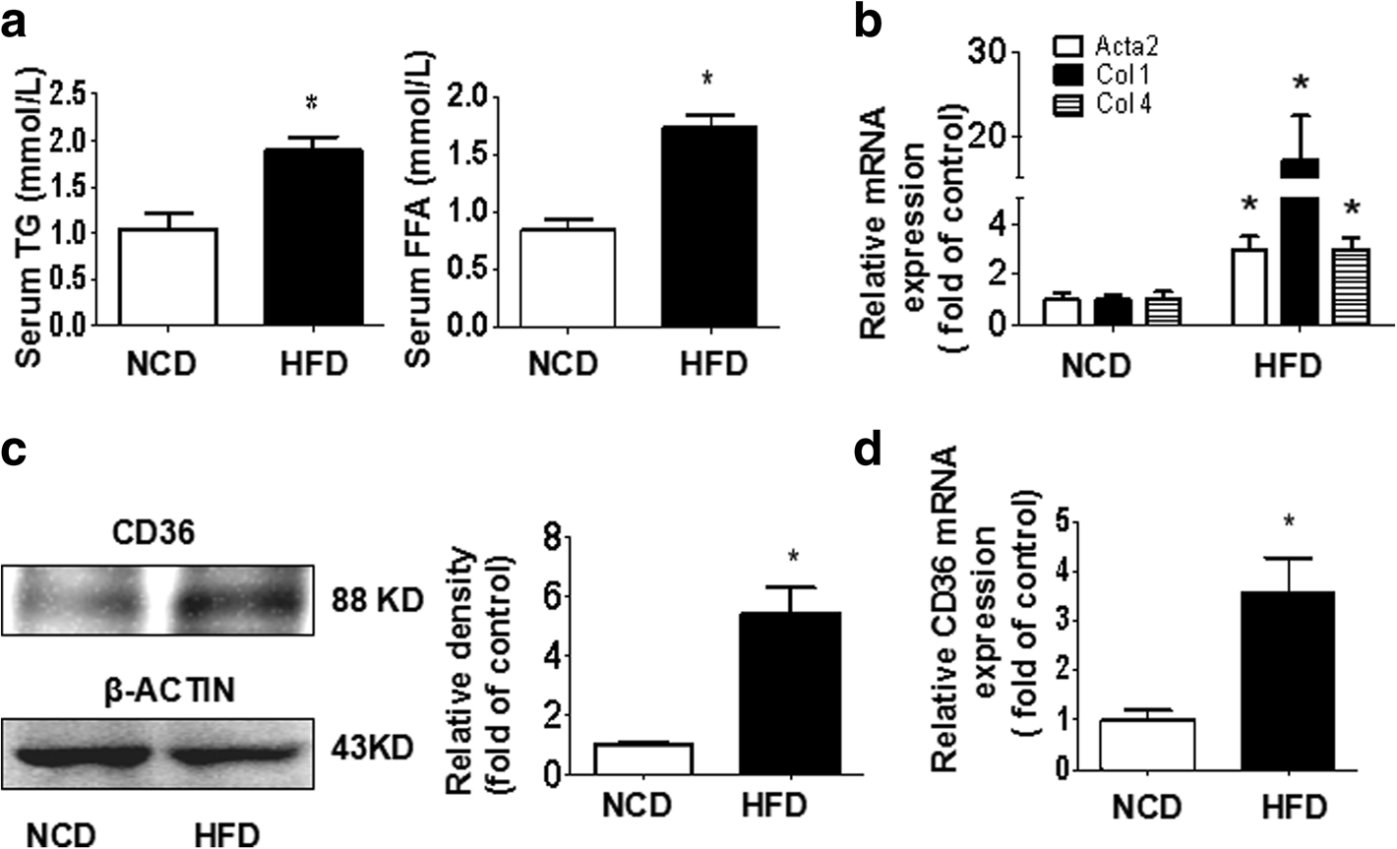
Effects of HFD on profibrogenic gene and CD36 expression in the livers of C57BL/6 J mice. Mice were fed a normal chow diet (NCD) or a high-fat diet (HFD) for 14 weeks. The levels of FFA and TG in serum were measured as described in the Materials and methods (**a**). The hepatic mRNA expression of *Acta2, Col 1,* and *Col 4* was determined by real-time PCR (**b**). The hepatic protein expression of CD36 was examined by western blotting (**c**). The hepatic mRNA expression of *CD36* was determined by real-time PCR (**d**). The results are depicted as the mean ± SEM, *P < 0.05 versus the NCD group

Serum FFA and TG levels were increased in HFD-fed mice compared with NCD-fed mice (Fig. 1a). Hepatic mRNA expression of markers of fibrogenesis, including *Acta2, Col 1,* and *Col 4,* was significantly up-regulated in HFD-fed mice (Fig. 1b). Additionally, we also found that HFD increased hepatic CD36 protein and mRNA expression (Fig. 1c, d).

### PA promotes hepatocyte activation and up-regulates CD36 expression

**Fig. 2:**
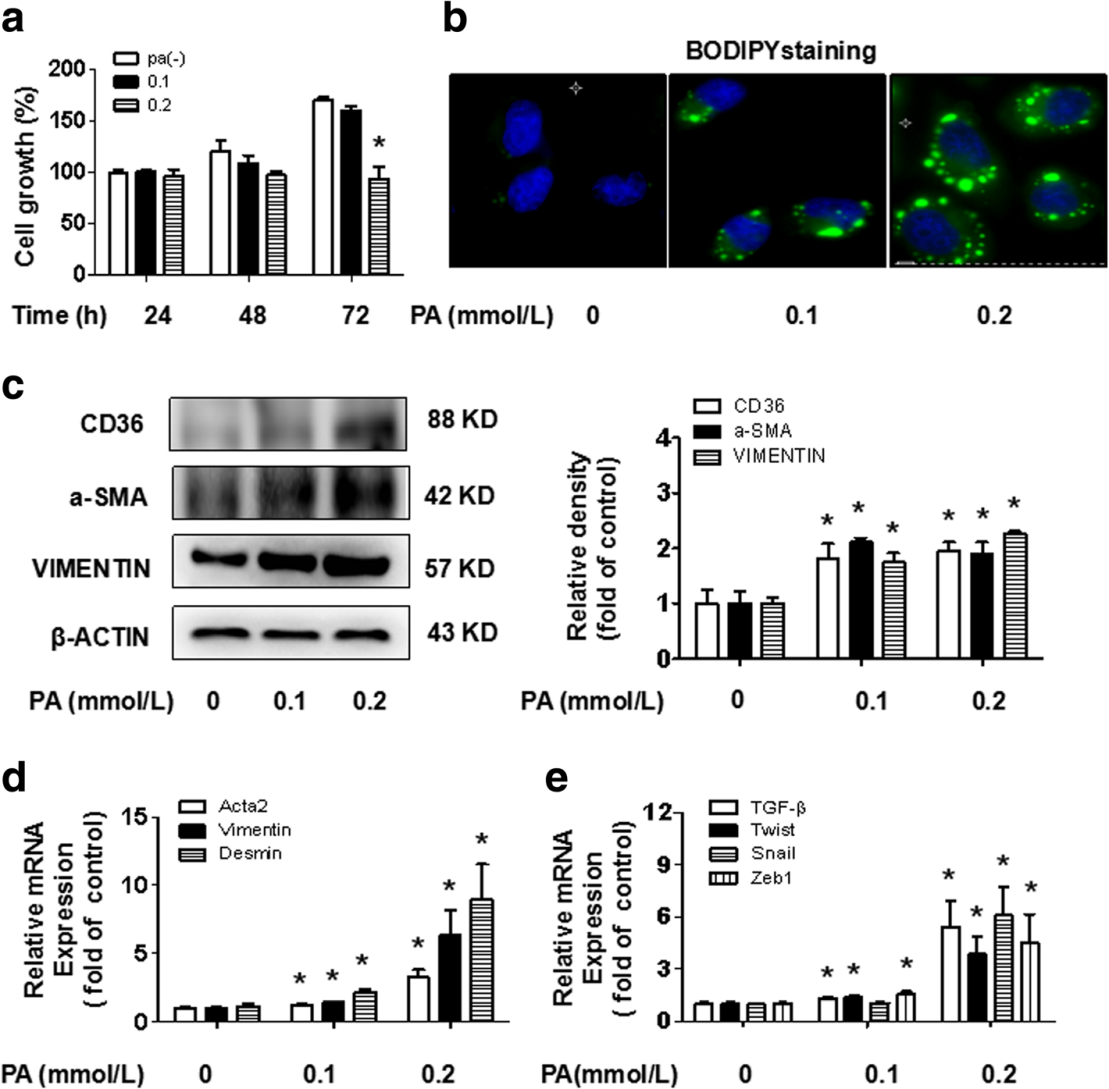
Effects of PA on hepatocyte activation and CD36 expression. Hepatocytes were incubated in serum-free medium containing different concentrations of PA. After the time indicated, a CCK-8 cell proliferation assay was performed to detect hepatocyte proliferation in response to PA (**a**). Lipid accumulation was observed by BODIPY staining (original magnification× 800, **b**). The protein expression of CD36, a-SMA, and VIMENTIN was examined by western blotting (**c**). The mRNA expression of *Acta2, Vimentin, Desmin* and TGF-β signaling pathway related genes *TGF-β, Snail, Twist,* and *Zeb1* was determined by real-time PCR (**d, e**). The results are depicted as the mean ± SEM, *P < 0.05 versus the 0 mmol/L PA group

Hepatocytes were treated with PA at different concentrations and times to determine the effect of PA on hepatocyte lipotoxicity and proliferation. We noticed that there was significant cytotoxicity at the 0.4 mmol/L concentration, but PA was comparatively non-toxic at the 0.1 mmol/L and 0.2 mmol/L concentrations (data not shown). Furthermore, there was no stimulatory effect of PA on hepatocyte proliferation (Fig. 2a). Following BODIPY staining, we explored the concentrations of PA that enhanced lipid accumulation in hepatocytes (Fig. 2b). We found that CD36 protein expression was increased in PA-treated hepatocytes at both 0.1 mmol/L and 0.2 mmol/L concentrations (Fig. 2c). Homoplastically, PA up-regulated the protein expression of a-SMA and VIMENTIN and the mRNA expression *of Acta2, Vimentin* and *Desmin* (Fig. 2d), which allowed hepatocytes to acquire an activated phenotype. Moreover, this trend was consistent with the mRNA expression of *TGF-β* and key downstream transcription factors S*nail*, *Twist* and Z*eb1* (Fig. 2e). These results suggest that PA mediates the up-regulation of CD36 expression and promotes hepatocyte activation.

### CD36 is involved in PA-induced hepatocyte activation

**Fig. 3:**
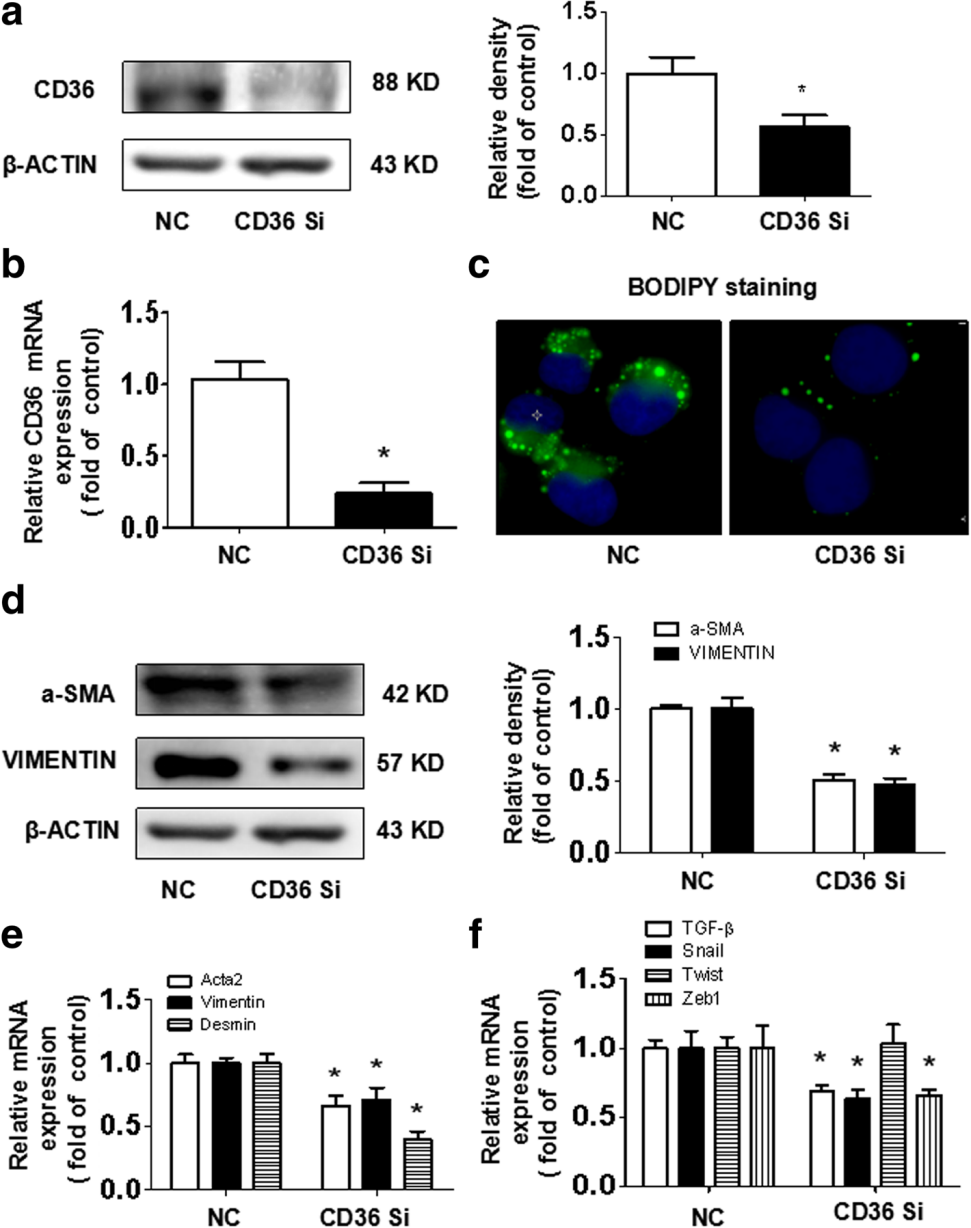
Effects of the suppression of CD36 on PA-induced hepatocyte activation. CD36-knockdown hepatocytes were treated with 0.2 mmol/L PA for 48 h. The protein and mRNA expression of CD36 were examined by western blotting (**a**) and real-time PCR (**b**). Lipid accumulation was observed by BODIPY staining (original magnification× 800, **c**). The protein expression of a-SMA and VIMENTIN were examined by western blotting (**d**). The mRNA expression of *Acta2, Vimentin, Desmin* and TGF-β signaling pathway related genes *TGF-β, Snail, Twist* and *Zeb1* were determined by real-time PCR (**e, f**). The results are depicted as the mean ± SEM, *P < 0.05 versus the NCD group

A PA concentration of 0.2 mmol/L has a marked effect on hepatocyte activation; therefore, we selected this concentration for additional experiments. We knocked down CD36 by transfecting CD36 siRNA into hepatocytes to confirm the role of CD36 in PA-induced hepatocyte activation. Our data demonstrated that CD36 siRNA transfection resulted in markedly decreased CD36 protein and mRNA expression in PA-induced hepatocytes by western blotting and real-time PCR (Fig. 3a, b), which suggested that the CD36 knockdown hepatocyte cell model has been established successfully. BODIPY staining showed that lipid accumulation clearly decreased in the CD36 knockdown group (Fig. 3c), suggesting that PA promotes hepatocyte lipid accumulation by CD36. Protein expression of activated hepatocyte markers a-SMA and VIMENTIN decreased in the CD36 knockdown group compared with the control group (Fig. 3d). Meanwhile, the mRNA expression of *Acta2, Vimentin, Desmin,* and TGF-β signaling pathway related genes, such as *TGF-β*, *Snail*, and *Zeb1*, were also down-regulated in the CD36 knockdown group (Fig. 3e, f). These findings suggest that PA promoted hepatocyte activation was mediated by CD36.

### CD36 mediates PA-induced hepatocyte activation via oxidative stress

**Fig. 4:**
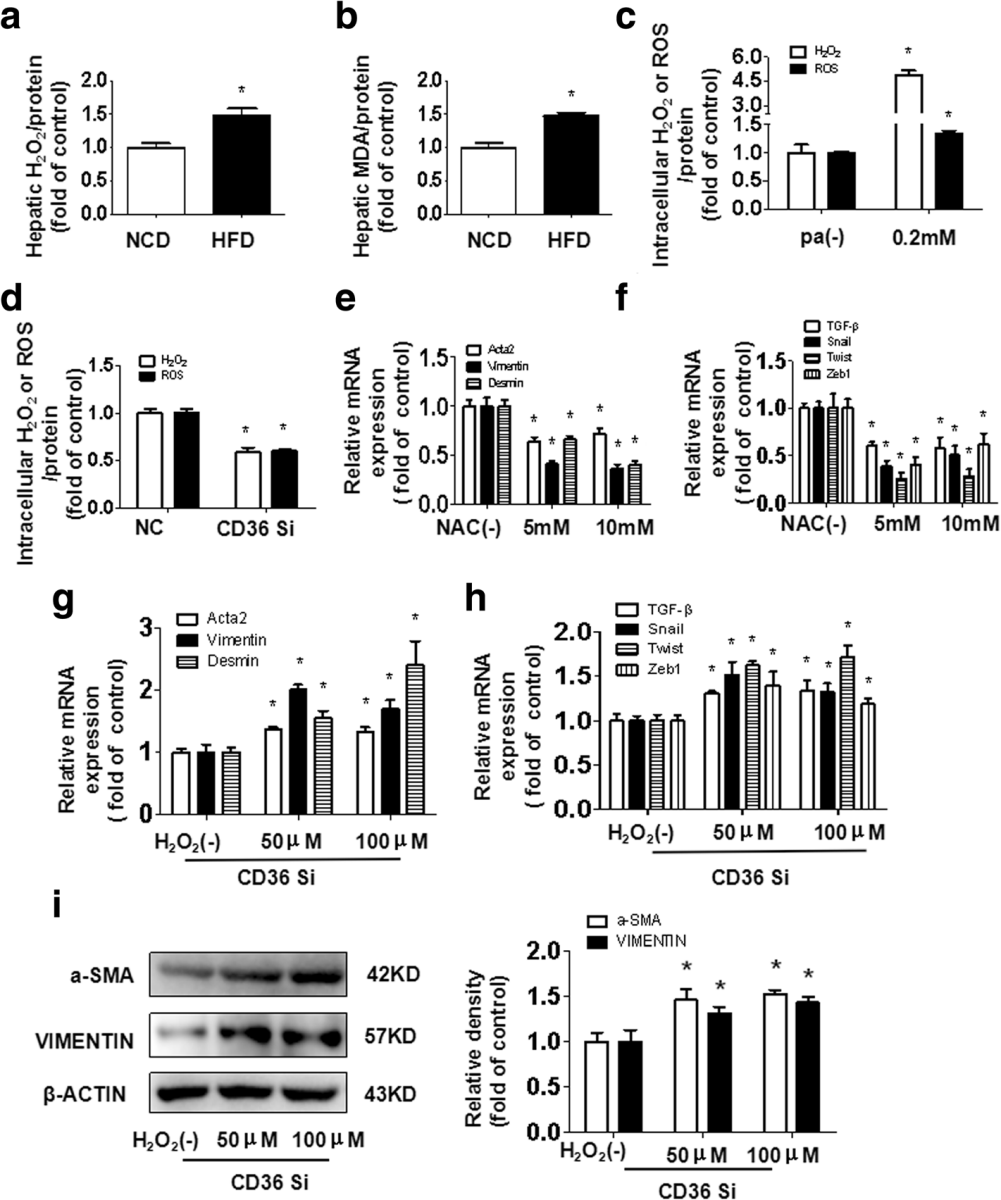
The oxidative stress involved in PA-induced hepatocyte activation mediated by CD36. H_2_O_2_ levels and MDA levels in the livers of mice fed an NCD or HFD for 14 weeks are shown (**a, b**). *P < 0.05 versus the NCD group. Hepatocytes were incubated in serum-free medium containing 0.2 mmol/L PA for 48 h, and H_2_O_2_ levels and ROS levels were then determined (**c**). *P < 0.05 versus the PA(−) group. CD36-knockdown hepatocytes were treated with 0.2 mmol/L PA; after 48 h, H_2_O_2_ levels and ROS levels were measured (**d**). *P < 0.05 versus the NC group. Hepatocytes were incubated in serum-free medium containing 0.2 mmol/L PA for 48 h alone or with pretreatment of NAC for 2 h. The mRNA expression of *Acta2, Vimentin, Desmin* and TGF-β signaling pathway related genes *TGF-β, Snail, Twist,* and *Zeb*1 were determined by real-time PCR (**e, f**). *P < 0.05 versus the NAC(−) group. CD36-knockdown hepatocytes were treated with 0.2 mmol/L PA for 48 h in the absence or presence of H_2_O_2_ for 24 h. The mRNA expression of Ac*ta2, Vimentin, Desmin* and TGF-β signaling pathway related genes *TGF-β, Snail, Twist,* and *Zeb*1 were determined by real-time PCR (**g-h**). The protein expression of a-SMA and VIMENTIN were examined by western blotting (**i**). *P < 0.05 versus the H_2_O_2_ (−) group. All results are depicted as the mean ± SEM

Oxidative stress has been shown to be involved in hepatotoxicity when pro-oxidative capacity overwhelms antioxidant capacity. As shown by our results, HFD-fed mice had greater H_2_O_2_ and MDA production in the liver than control mice (Fig. 4a, b). Similarly, the production of H_2_O_2_ and ROS was considerably higher in hepatocytes treated with PA than in the control group (Fig. 4c). Knockdown of CD36 considerably decreased the production of H_2_O_2_ and ROS in hepatocytes treated with PA (Fig. 4d). These results suggested that the level of oxidative stress depends on hepatocyte CD36 expression when treated with PA. We individually applied the antioxidant NAC in PA-treated hepatocytes and H_2_O_2_ in PA-treated hepatocytes with CD36 knockdown to determine whether the oxidative stress mediated by CD36 was involved in PA-induced hepatocyte activation. We further found that NAC significantly decreased PA-induced hepatocyte activation and TGF-β signaling pathway related genes (Fig. 4e, f). In addition, the H_2_O_2_ supplement largely abrogated the improved effect of CD36 knockdown on hepatocyte activation (Fig. 4g-i). These data suggest that oxidative stress is critical in PA-induced hepatocyte activation and that this activation is mediated by CD36.

## Discussion

The purpose of our current study was to clarify the effects of lipids on hepatocyte activation. Here, we show that: 1) LCFA treatment induced hepatocyte activation, evidenced by up-regulated expression of Acta2, Vimentin, Desmin*,* and TGF-β signaling pathway; 2) CD36 inhibition attenuated LCFA-induced hepatocyte activation; 3) Oxidative stress was critical in LCFA-induced hepatocyte activation, which was mediated by CD36.

Many studies have demonstrated the potential role of functional food ingredients in controlling dyslipidaemia and lipid metabolism both in animal models and in humans [20]. Excess lipids intake induces hyperlipidaemia and lipid accumulation in the liver that results in the development of NAFLD [21]. Previous studies have suggested a close association between lipids and liver fibrosis. Increased cholesterol intake accelerated liver fibrosis, which was mainly due to increased free cholesterol accumulation in HSCs, thus promoting HSC activation [14]. Previous studies also indicated that LCFA and ox-LDL had profibrogenic effects on HSCs via the up-regulation of ECM synthesis [15, 19]. Numerous studies have demonstrated that hepatocytes are important cells when they evolve into an activated phenotype in the development of hepatic fibrosis over decades [11, 22]. Although there are reports that FFA has a certain effect on HSC activation [15], there is a relatively poor understanding of the FFA involved in hepatocyte activation. In this study, we demonstrated that PA induces hepatocyte activation, as evidenced by increased mRNA abundance of *Acta2, Vimentin* and *Desmin*, which are representative markers of hepatocyte activation.

The activation of TGF-β signaling pathways has been verified, and this activation could promote the subsequent activation of EMT transcription factors, including SNAIL, ZEB and TWIST, which allows epithelial cells to acquire a mesenchymal phenotype [23]. In particular, it is now accepted that TGF-β is a crucial fibrogenic mediator in the induction of hepatocyte activation, which plays a significant role in the perpetuation of hepatic fibrosis [24]. Our data demonstrated PA increased the mRNA expression of TGF-β signaling pathway related gene expression, including *TGF-β*, *Snail*, and *Zeb1*, and these genes were down-regulated in the CD36 knockdown hepatocytes. Our results suggest that PA may be involved in the regulation of hepatocyte activation through TGF-β related pathways, which may be mediated by CD36.

Previous studies have revealed that oxidative stress is closely related to TGF-β signaling pathway in liver fibrogenesis [25]. It has been reported that the superabundant accumulation of lipids in hepatocytes could exceed the oxidative capacity of metabolism, causing oxidative stress [26], and CD36 may participate in this process. CD36 is an important mediator of the production of ROS and oxidative stress [27], besides regulation of hepatic fatty acid uptake and TG storage, it also plays a pathological role in the development of various diseases [17]. So, in the present study, we sought to determine whether CD36 is involved in PA-induced hepatocytes activation through oxidative stress. Our data showed that PA or HFD significantly increased the level of oxidative stress in hepatocytes or C57BL/6 J mice and that these phenotypes were reversed after knockdown of CD36 in hepatocytes. In addition, N-acetylcysteine, an antioxidant, largely inhibited the hepatocyte-activating effects of PA. Furthermore, supplementation of H_2_O_2_ markedly abrogated the improved effects of CD36 knockdown on hepatocyte activation. Taken together, our data suggest that oxidative stress is critical in PA-induced hepatocyte activation and that this activation is mediated by CD36.

Currently, increasing attention has been paid to the involvement of CD36 in liver diseases. In fact, CD36 has been associated with obesity and diabetes in human diseases; Particularly, increased expression of hepatic CD36 is closely related to the development of NAFLD and non-alcoholic steatohepatitis (NASH) with insulin resistance [28]. Moreover, our previous work has demonstrated that CD36 plays an important role in balancing hepatic ROS and regulating macrophage infiltration, which could be a new potential therapeutic strategy to prevent NASH development [29]. Hence, it is believed that CD36 is emerging as a novel target for chronic liver disease.

## Conclusions

Our findings demonstrated that PA induces the production of oxidative stress, promoting hepatocyte activation, thus playing an imperative role in the process of hepatic fibrosis. Furthermore, we have uncovered, for the first time, the molecular mechanisms involved in hepatocyte activation via oxidative stress were mediated by CD36, which could be used to acquire novel targets for the prevention and treatment of hepatic fibrosis and may provide a new entry point for researchers studying hepatic fibrosis.

## Abbreviations

BSA: Bovine serum albumin
CD36: Cluster of differentiation
Col: Collagen type
ECM: Extracellular matrix
EMT: Epithelial-mesenchymal transition
FFA: Free fatty acid
H_2_O_2_: Hydrogen peroxide
HFD: High fat diet
HSCs: Hepatic stellate cells
LCFA: Long-chain fatty acid
MDA: Malondialdehyde
NAC: N-acetylcysteine
NAFLD: Non-alcoholic fatty liver disease
NASH: Non-alcoholic steatohepatitis
NCD: Normal chow diet
Ox-LDL: Low-density lipoprotein
PA: Palmitic acid
ROS: Reactive oxygen species
TC: Total cholesterol
TG: Triglyceride
TGF-β: Transforming growth factors-β
